# Co-expression of nuclear P38 and hormone receptors is prognostic of good long-term clinical outcome in primary breast cancer and is linked to upregulation of DNA repair

**DOI:** 10.1186/s12885-018-4924-2

**Published:** 2018-10-23

**Authors:** Simon J. Johnston, Dena Ahmad, Mohammed A. Aleskandarany, Sasagu Kurozumi, Chris C. Nolan, Maria Diez-Rodriguez, Andrew R. Green, Emad A. Rakha

**Affiliations:** Nottingham Breast Cancer Research Centre, Division of Cancer and Stem Cells, School of Medicine, The University of Nottingham, Nottingham, UK

**Keywords:** Breast cancer, P38, MAPK, Oestrogen receptor, Adjuvant, Survival, Prognosis

## Abstract

**Background:**

P38 mitogen activated protein kinase is an intermediary signal transduction factor with context-specific roles in breast cancer. Recent mechanistic studies add to the growing consensus that P38 is a tumour suppressor, and it may represent a novel target for breast cancer treatment. The aim of this study is to add definitive data on the prognostic value of P38 and its link with biomarkers in primary breast cancer.

**Methods:**

A large, well-characterised series of 1332 primary breast cancer patients with long-term clinical follow-up was assessed for P38 expression by immunohistochemistry. Association of clinicopathological factors and a panel of breast cancer biomarkers was determined by chi-squared test, and multivariate survival analysis was performed using Cox Proportional Hazards regression modelling.

**Results:**

This study shows that nuclear P38 is co-expressed with nuclear hormone receptors (*p* < 0.001) and is an independent prognostic marker of good long-term clinical outcome in primary breast cancer (hazard ratio 0.796, 95% confidence interval 0.662–0.957, *p* = 0.015). Significant association was found between expression of P38 and markers of DNA repair including nuclear BRCA1 and RAD51, and cleaved PARP1 (all *p* < 0.001).

**Conclusions:**

The findings support the proposed role for P38 as a tumour suppressor in breast cancer via upregulation of DNA repair proteins and provide novel hypothesis-generating information on the potential role of P38 in adjuvant therapy decision making.

**Electronic supplementary material:**

The online version of this article (10.1186/s12885-018-4924-2) contains supplementary material, which is available to authorized users.

## Background

Adjuvant treatment of breast cancer is determined primarily by tumour biology. P38 mitogen activated protein kinase (MAPK) has been proposed as a potential target for the adjuvant treatment of breast cancer, but its prognostic value in this context has yet to be defined.

P38 MAPKs are essential components of intracellular signal transduction. Cancer cells respond to extracellular stimuli such as pro-inflammatory cytokines and oxidative stress via MAPK pathways mediated by ERK, JNK, and p38 protein kinase activation [1, 2]. Once phosphorylated, P38 in cytoplasm translocates to the nucleus where the signal is cascaded by phosphorylation of protein kinases and transcription factors (TFs) [3]. Integration of the cellular signal by downstream TFs such as cyclic-AMP dependent TF2 (ATF2) and TP53 determines gene expression programmes that ultimately dictate phenotypic response [4–6].

In 70% of breast cancer, the driving TF is oestrogen receptor (ER) [7]. Along with progesterone receptor (PR), expression of ER defines luminal breast cancer and is associated with good prognosis and response to endocrine therapy [8]. The role of P38 as an intermediary signal transduction factor in this context is complex, and evidence of its prognostic value is mixed [9–12].

Emerging mechanistic data add to a growing consensus that P38 is a tumour suppressor in breast cancer [5, 13–15]. These data may have important clinical implications, for example in defining the clinical context in which small molecule inhibitors of P38 may have a role in breast cancer treatment. However, to date, clinical cohorts investigating the role and prognostic value of P38 in breast cancer have been limited in size (*N =* 45 to 335) and conclusions are inconsistent between the studies [16–18]. To determine the context-specific roles of P38 in breast cancer, e.g. by molecular subtype (luminal, HER2 positive or triple negative), large clinical cohort studies are required.

The aim of this study is to address the need for definitive data on the co-expression of P38 with key clinicopathological markers including ER and HER2 and its association with clinical outcome. Data from this large (*N =* 1332), clinically and molecularly annotated dataset with long-term clinical follow-up (median 166 months, range 1–308 months) is interpreted alongside current mechanistic data on P38 in breast cancer and the Molecular Taxonomy of Breast Cancer International Consortium (METABRIC, *N =* 1980) external dataset (accession number EGAS00000000098).

The study shows that co-expression of nuclear phosphorylated P38 (p-P38) and nuclear hormone receptors ER and / or PR is associated with good long-term clinical outcome in primary breast cancer. In support of the emerging consensus that P38 is a tumour suppressor, the current study also demonstrates that nuclear p-P38 is an independent marker of good prognosis in all breast cancer subtypes. In support of a recent finding that P38 positively regulates DNA repair [15], the current study shows that nuclear p-P38 is associated with nuclear expression of DNA repair markers BRCA1 and RAD51, and cleaved PARP1. These findings have important implications for breast cancer prognostics and for the clinical application of P38 inhibitors.

## Methods

This was a retrospective study of 1339 primary invasive BC patient samples within a previously constructed tissue microarray (TMA), as previously described [19, 20]. All patients were treated in Nottingham University Hospital NHS Trust between 1989 and 1998. The average age of patients was 54 years (range 18–71). Clinical and pathological information were available for patients including patients’ age, tumour histological grade, tumour size, stage, vascular invasion (VI) and Nottingham Prognostic Index (NPI) (Table 1). All patients were treated with adjuvant therapy according to their NPI score and ER status. NPI score is determined by size, grade and stage of tumour and is used to stratify patients into risk categories. If NPI was ≤3.4, prognosis is considered good to excellent (85% 5-year survival), and no adjuvant treatment was given. Cases with NPI > 3.4 have moderate prognosis (70% 5-year survival), and systemic adjuvant therapy was given; endocrine therapy in ER positive (tamoxifen or aromatase inhibitor, with the addition of goserelin if the patient was premenopausal), while in ER-negative, systemic chemotherapy was given in the form of cyclophosphamide, methotrexate and 5-fluorouracil (CMF). Outcome data were collected on a prospective basis.

**Table 1:** Patient characteristics (*N =* 1332)

Variable		number	%
Age (years)	< 50	454	34.2%
	≥50	874	65.8%
Tumour size (mm)	< 20	646	48.7%
	≥20	681	51.3%
Nodal stage	1	810	60.8%
	2	406	30.5%
	3	111	8.3%
Grade	1	202	15.2%
	2	459	34.5%
	3	666	50.2%
ER status	negative	314	23.8%
	positive	1005	76.2%
HER2 status	negative	1102	86.7%
	positive	169	13.3%
Vascular invasion	negative	733	55.0%
	suspected (probable)	153	11.5%
	positive (definite)	435	32.7%
Distant metastasis	negative	826	62.5%
	positive	496	37.5%

Overall survival (OS) was defined as the time in months from the date of surgery to death from any cause, at a median follow up of 166 months. Table 1 summarises the main clinicopathological criteria of the patient cohort.

### Immunohistochemistry

From TMA blocks, 4 μm sections were cut onto Xtra slides, which were heated to 60 °C for 10 min and deparaffinised using xylene and rehydrated using grades alcohol series and tap water. Microwave antigen retrieval (Sharp R-254 M 800 W) was used for 20 min to heat slides with 0.01 M citrate buffer (pH = 6). Immunohistochemistry (IHC) was conducted using Novocastra Novolink Polymer Detection System (Code: RE7280-K, Leica Microsystems, Newcastle, UK) following the manufacturer instructions. Primary antibodies were used at the following conditions. Rabbit anti human P38 antibody (Cell Signalling, 1:50) and rabbit antihuman phosphorylated-P38 (p-P38, Cell Signalling, 1:200) were incubated for 1 h at room temperature or overnight at 4 °C, respectively. Slides were dehydrated in alcohol and cleared in xylene using Leica Autostainer, then mounted using DPX (BDH, Poole, UK).

Antibody specificity was confirmed by Western blot using MCF7 cell line lysate. In brief, MCF7 breast cancer cell line was lysed in RIPA buffer (Sigma Aldrich, UK) supplemented with 1X protease inhibitor (Sigma Aldrich, UK) and 1X phosphatase inhibitor (Sigma Aldrich, UK). Protein lysates were mixed with 4x NuPage LDS sample buffer (Invitrogen) and 10 X NuPage reducing solution. Samples were then loaded on (4–12%) SDS-PAGE precast gel (Novex, Life technologies), blotted on Hybond ECL nitrocellulose membrane (GE, Healthcare), then blocked with 5% non-fat dried milk before incubation with P38 antibody (1:1000) or p-P38 antibody (1:1000) for 1 h at room temperature. HRP anti-rabbit antibody (1:200, DAKO) was used before developing the membrane using ECL detection reagent (Amersham, UK) and exposing the membrane to X-ray film (Kodak, Sigma Aldrich, UK) for final detection of the protein bands. Single bands resulted with both antibodies at the expected molecular weights (Additional file 1: Figure S1).

### IHC scoring and statistical analysis

All TMA slides were scanned at 200X magnification and scored visually using NanoZoomer software (Hamamatsu Photonics, Welwyn Garden City, UK). Both the staining intensity (negative, low, moderate and high) and percentage of staining were assessed, and a final H-score was calculated per sample. The H score were dichotomised into two main categories (high and low) using a cut-point value obtained using X-tile bioinformatics software [21].

Chi-squared (Χ^2^) test was used to study the association between the categorised data of P38/p-P38 and clinicopathological criteria. Kaplan–Meier was used to plot survival curves of OS and Log-Rank tests were used to estimate their significance. Cox multivariate analysis was performed using the log-rank test. A two-tailed *p* value < 0.05 was considered significant. Statistical analyses were performed using SPSS 21 statistical software (SPSS IBM Corp, Chicago, USA) and reported in line with REMARK guidelines [22].

## Results

### Expression of pan-P38 and phosphorylated (p)-P38

Tissue expression of pan-P38 and phosphorylated P38 (p-P38) was assessed in an invasive breast cancer TMA. Pan-P38 was expressed in the cytoplasm of invasive breast cancer cells with different staining intensities observed (Additional file 1: Figure S2, A-C). Phosphorylated P38 expression was mainly nuclear with weak cytoplasmic p-P38 staining across the different cores observed (Additional file 1: Figure S2, D-F).

A cut-off value of 110 was used with pan-P38 with 60.3% (*N =* 703) showing negative/low expression compared to 39.7% of cases with positive/high expression. The cut-points of cytoplasmic and nuclear p-P38 H-scores were 80 and 110, respectively. Cytoplasmic and nuclear p-P38 expression was negative/low in 68.5% (*N =* 914) and 70.5% (*N =* 941) respectively, and positive/high in 31.5% (*N =* 419) and 29.5% (*N =* 394), respectively.

### Association of P38 with clinicopathological variables and disease biomarkers

Pan-P38 and nuclear p-P38 expression were significantly associated with positive expression of nuclear hormone receptors ER and PR, which define luminal disease (see Table 2). Neither pan-P38 nor nuclear p-P38 expression was associated with HER2 expression. There was positive correlation of both pan-P38 and nuclear p-P38 with phosphorylated ATF2, a transcription factor downstream of ERK, JNK and P38 kinases (both *p < 0.*001, data not shown). For clarity, further analysis in this study is restricted to factors related exclusively to P38, independently of ERK and JNK. Cytoplasmic staining of p-P38, which was globally weaker in intensity, did not validate the associations seen with pan-P38 or nuclear p-P38, with the exception of stage (data not shown). Cytoplasmic p-P38 was associated with negative expression of ER. Nuclear p-P38 was associated with a greater number of favourable prognostic variables, with greater statistical confidence (i.e. lower *p* values). Therefore, nuclear p-P38 was taken forward as a more sensitive candidate marker of clinicopathological features and clinical outcome. Pan-P38 is eliminated from further analysis and discussion. Nuclear p-P38 is hereafter referred to as ‘p-P38’.

**Table 2:** Activated P38 is associated with luminal phenotype (ER &/or PR expression) and favourable clinicopathological prognostic indicators (*N =* 1332)

Variable		pan-P38					nuclear p-P38				
		Negative/low		Positive/high		*p*	Negative/low		Positive/high		*p*
Age	< 50 yrs	187	57.9%	136	42.1%	0.258	318	70.0%	136	30.0%	0.751
	≥50 yrs	363	61.7%	225	38.3%		620	70.9%	254	29.1%	
Size	< 20 mm	232	56.2%	181	43.8%	**0.025** ^*^	421	65.2%	225	34.8%	**< 0.001** ^*^
	≥20 mm	316	63.7%	180	36.3%		516	75.8%	165	24.2%	
Stage	1	334	60.5%	218	39.5%	0.890	550	67.9%	260	32.1%	**0.007** ^*^
	2–3	214	59.9%	143	40.1%		387	74.9%	130	25.1%	
Grade	1–2	237	55.4%	191	44.6%	**0.004** ^*^	413	62.5%	248	37.5%	**< 0.001** ^*^
	3	311	64.7%	170	35.3%		524	78.7%	142	21.3%	
NPI	good / medium	440	58.9%	307	41.1%	0.095	760	68.8%	344	31.2%	**0.002** ^*^
	Poor	109	66.1%	56	33.9%		179	79.2%	47	20.8%	
VI	negative or suspected	354	59.9%	237	40.1%	0.722	602	67.9%	284	32.1%	**0.002** ^*^
	positive (definite)	192	61.1%	122	38.9%		331	76.1%	104	23.9%	
ER	Negative	167	74.6%	57	25.4%	**< 0.001** ^*^	244	77.7%	70	22.3%	**< 0.001** ^*^
	Positive	379	55.3%	306	44.7%		690	68.7%	315	31.3%	
PR	Negative	265	72.4%	101	27.6%	**< 0.001** ^*^	398	76.5%	122	23.5%	**< 0.001** ^*^
	Positive	264	51.4%	250	48.6%		507	67.3%	246	32.7%	
HER2	Negative	463	60.5%	302	39.5%	1.000	775	70.3%	327	29.7%	0.237
	Positive	66	61.1%	42	38.9%		127	75.1%	42	24.9%	

*NPI* Nottingham Prognostic Index, *VI* vascular invasion

^*^p value in bold type indicates statistical significance defined as *p* < 0.05 by Χ^2^ test

### Association of p-P38 with DNA repair markers

**Fig. 1:**
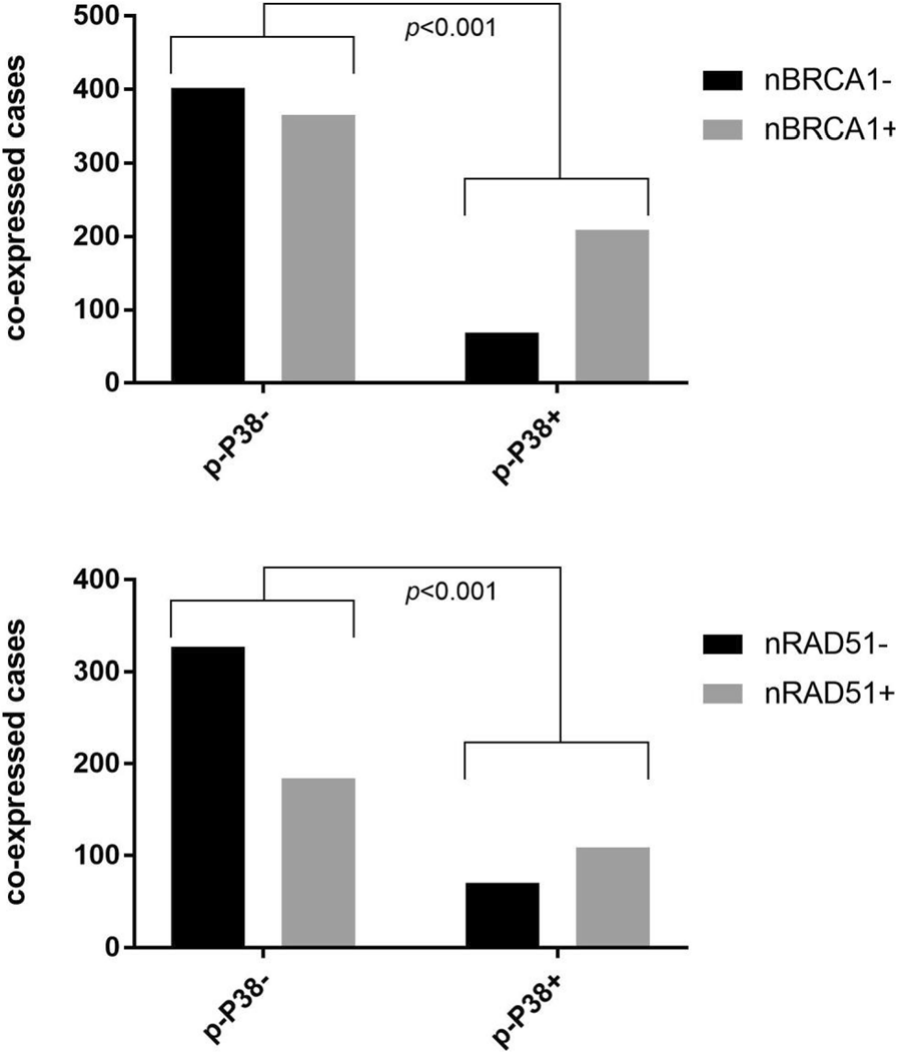
Nuclear BRCA1 (nBRCA1) and RAD51 (nRAD51) positivity is enriched in tumours expressing high levels of nuclear phosphorylated P38 (p-P38+) (*p* < 0.001 by Χ2 test)

There was significant positive association of p-P38 with nuclear BRCA1 and RAD51 (see Table 3 and Fig. 1). There was also enrichment of positive cleaved PARP1 expression in cases expressing high p-P38, although the majority of cases were positive for cleaved PARP1 irrespective of p-P38 status (Table 3). No such association was found between p-P38 and the transcription factor TP53.

**Table 3:** Nuclear p-P38 is associated with DNA repair markers

Variable		nuclear p-P38				
		negative / low		high		*p*
nuclear BRCA1	negative	402	(85.4%)	69	(14.6%)	**< 0.001** ^*^
	positive	365	(63.6%)	209	(36.4%)	
nuclear BRCA2	negative	540	(72.0%)	210	(28.0%)	0.393
	positive	44	(66.7%)	22	(33.3%)	
PARP1	negative	346	(75.4%)	113	(24.6%)	0.074
	positive	373	(70.1%)	159	(29.9%)	
cleaved PARP1	negative	114	(85.7%)	19	(14.3%)	**< 0.001** ^*^
	positive	563	(71.5%)	224	(28.5%)	
nuclear RAD51	negative	327	(82.4%)	70	(17.6%)	**< 0.001** ^*^
	positive	184	(62.8%)	109	(37.2%)	
TP53	negative	636	(70.6%)	265	(29.4%)	0.337
	positive	268	(73.4%)	97	(26.6%)	

^*^p value in bold type indicates statistical significance (*p* < 0.05, by Χ^2^ test)

### Overall survival analysis based on clinicopathological characteristics, hormone receptor status and P38 expression

**Fig. 2:**
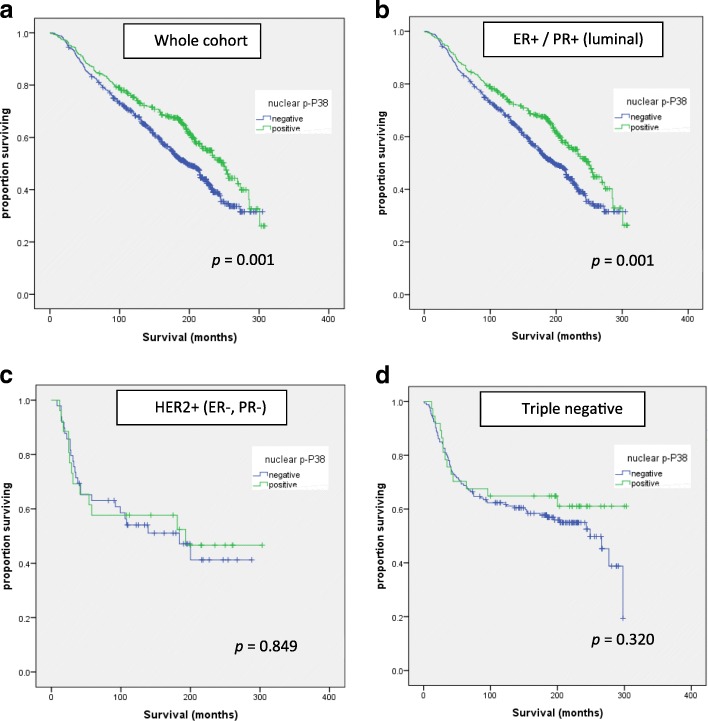
Overall survival by p-P38 status; Nuclear p-P38 is prognostic of overall survival in (**a**) the whole cohort and in (**b**) the luminal subtype, with identical statistical significance by log-rank test. There was no difference in overall survival in (**c**) the HER2+ or (**d**) the triple negative subgroup by p-P38 status

Kaplan-Meyer plots of OS by p-P38 status show that the prognostic significance of P38 is identical in the luminal subtype (ER and/or PR positive, including HER2 positive cases) to the whole cohort (*p* = 0.001). No significant association with survival was found in the smaller HER2 positive (ER and PR negative) or triple negative subtypes (see Fig. 2).

Nuclear p-P38, which is associated with expression of hormone receptor biomarkers of endocrine therapy response, ER and PR (Table 2), is an independent prognostic marker of good clinical outcome in multivariate analysis (Table 4). Multivariate analysis was performed on NPI to avoid overlap between the clinicopathological variables, e.g. size and stage. In ER+/PR+ (luminal) disease, median overall survival was 250 months in tumours expressing high nuclear p-P38 versus 197 months for p-P38 negative cases (*p* = 0.001) (see Fig. 2b).

**Table 4:** Overall survival analysis based on clinicopathological characteristics, biomarker status and P38 expression

Characteristic		Univariate analysis			Multivariate analysis		
		HR	95% CI	*p*	HR	95% CI	*p*
p-P38	low	*reference*			*reference*		
	high	0.735	0.617–0.875	**0.001** ^*^	0.796	0.662–0.957	**0.015** ^*^
NPI	good/medium	*reference*			*reference*		
	poor	2.465	2.054–2.959	**< 0.001** ^*^	2.120	1.731–2.596	**< 0.001** ^*^
VI	negative or suspected	*reference*			*reference*		
	positive (definite)	1.436	1.225–1.683	**< 0.001** ^*^	1.161	0.973–1.384	0.097
ER	negative	*reference*			*n/a*		
	positive	0.917	0.764–1.100	0.350			
PR	negative	*reference*			*reference*		
	positive	0.711	0.607–0.832	**< 0.001** ^*^	0.830	0.703–0.980	**0.028** ^*^
HER2	negative	*reference*			*reference*		
	positive	1.722	1.393–2.128	**< 0.001** ^*^	1.486	1.191–1.856	**< 0.001** ^*^

*HR* hazard ratio for death (all causes), *CI* confidence interval, *ER/PR* oestrogen/progesterone receptor status (by immunohistochemistry)

^*^p value in bold type indicates statistically significant results (*p* < 0.05). NPI = Nottingham Prognostic Index; VI = vascular invasion; p-P38 = nuclear phosphorylated P38

From the whole cohort of 1332 patients, 288 were also included in the METABRIC dataset [23]. Analysis was performed on the association between both pan-P38 and p-P38 protein expression and mRNA expression of MAPK14, the gene encoding the main P38 isoform in breast cancer, P38α. There was no significant association between protein and mRNA expression, using median MAPK14 mRNA expression as a cut-off, by Χ^2^ test (*p* = 0.77), and no significant link with overall survival (HR 0.80, 95% CI 0.55–1.17; *p* = 0.25). Furthermore, there was no significant link between MAPK14 expression and overall survival in the complete METABRIC dataset (HR 1.10, 95% CI 0.96–1.26; *p* = 0.16).

**Fig. 3:**
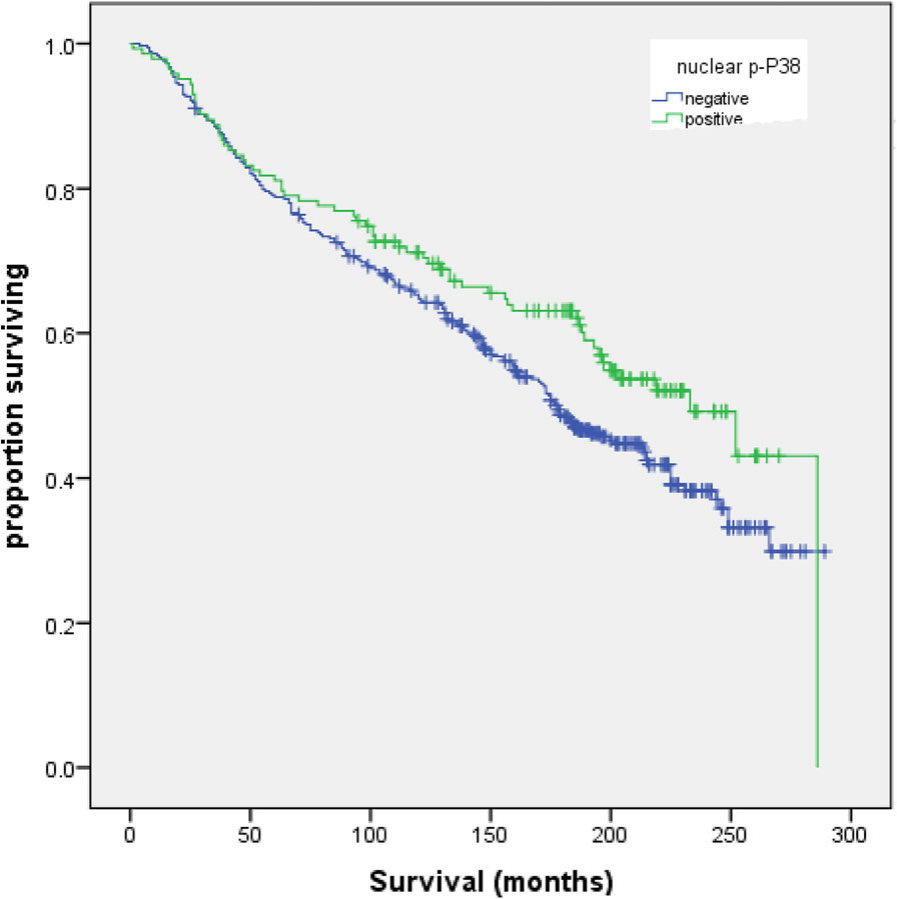
Overall survival after endocrine therapy by p-P38 status; Activation of P38 predicts better long-term outcome in patients treated with up to 5 years of adjuvant endocrine therapy

In patients who received endocrine therapy (ET), i.e. those with ER+/PR+ disease and NPI ≥ 3.4, overall survival was compared by nuclear p-P38 expression. Overall survival for patients who had ET was 233 months for patients with high nuclear p-P38 expressing tumours, versus 176 months in p-P38 negative cases (*p* = 0.043) (Fig. 3).

## Discussion

There is an emerging consensus, based mainly on mechanistic studies, that P38 is a tumour suppressor in breast cancer. Such reports have the potential to direct future clinical studies, but only if the true prognostic value of the factor of interest is known. Large clinical datasets are required to define the prognostic role of P38 in breast cancer and its subtypes with statistical confidence, and thereby provide essential supportive data for the conclusions of mechanistic studies and their clinical implications.

Results from the current study provide definitive evidence from a large clinical series (*N =* 1332) that nuclear activated (phosphorylated) P38 (p-P38) is co-expressed with nuclear hormone receptors (ER and PR), and that the co-expression of p-P38 with ER and/or PR is prognostic of good long-term clinical outcome. Nuclear p-P38 is an independent prognostic marker of good long-term clinical outcome and is associated with nuclear expression of DNA repair markers BRCA1 and RAD51. Interpreted alongside existing mechanistic studies, these data have important clinical implications.

### P38 as a tumour suppressor

Despite some studies supporting context-specific roles for P38, for example in tamoxifen and HER2-targeted therapy resistance, there is mounting evidence that in primary breast cancer P38 has a tumour suppressive effect [5, 13–15].

Previous studies into the prognostic value of P38 in breast cancer have been conducted on series of limited size, or in a specific subgroup or clinical context. For example, expression of phosphorylated P38 (p-P38) was assessed by IHC in 96 lymph node positive breast cancer patients [9]. High p-P38 expression was not significantly associated with overall survival in the overall cohort. Subgroup analysis of patients with highly proliferative tumours (measured by Ki67 expression) indicated that P38 was a poor prognostic marker; however, this was not confirmed using multivariate analysis.

The current study provides definitive evidence that p-P38 is an independent prognostic marker of good long-term clinical outcome in primary breast cancer. This supports the consensus that P38 is a tumour suppressor in breast cancer and thereby substantiates the clinical implications of key mechanistic studies on P38. This supports their translation into further clinical study.

For example, a recent report by Canovas et al. [15] highlights that breast tumour progression relies on P38 signalling in epithelial cells. The purported mechanism is that P38 protects breast cancer cells by positively regulating DNA repair. This prevents cell death secondary to the accumulation of DNA damage and chromosomal instability, and thereby inhibits any selective advantage of tumour cells that acquire mutations [24]. This supports the function of P38 as a tumour suppressor, and the mechanism is substantiated by the link between p-P38 and DNA repair markers including nuclear BRCA1 and RAD51 in the current study. This raises the possibility of P38-mediated upregulation of DNA repair in response to cellular stress via activation of the known BRCA1/RAD51 mechanism [25]. However, it also provides the rationale for combining taxane-based adjuvant chemotherapy with small molecule inhibition of P38: to increase DNA damage and chromosome instability by destabilising DNA repair mechanisms, and thereby increase the anti-tumoral response to chemotherapy.

There is also emerging evidence that P38 has a role in regulation of stromal expansion, thereby inhibiting breast cancer metastasis [14]. Evidence that P38 is tumour suppressive in primary breast cancer suggests that P38 inhibitors do not have a role as sole adjuvant therapy in primary breast cancer.

### Co-expression of P38 and nuclear hormone receptors ER and PR

The molecular function of P38 in solid tumours is thought to be cell-type specific [6]. Therefore, the role of P38 may vary according to the tissue of origin of a particular cancer, or clinicopathological features such as breast cancer subtype (e.g. luminal, HER2 positive, basal). However, existing evidence of co-expression of P38 with key biomarkers such as ER and HER2 is derived from small clinical cohorts, with inconsistency between the cohorts. As examples, one study (*N =* 45) found a significant positive association between protein expression of P38 and ER [16]. However, in another study (*N =* 355), expression of p38 was associated with HER2 and PR, but not ER expression [18]. Another cohort (*N =* 140) found that P38 was significantly associated with ER expression, but only if HER2 was co-expressed [17].

In the current study P38 activation, i.e. nuclear p-P38, was associated with ER and PR expression, with high statistical significance. However, no association was found between p-P38 and HER2 expression. Analysis by breast cancer subtype revealed that high P38 expression was significantly linked to a survival advantage in luminal disease but not in HER2 positive (ER and PR negative) or triple negative disease. To the best of our knowledge, our study has the largest sample size investigating the link between P38 and clinical biomarkers such as ER, PR and HER2.

The link between nuclear p-P38 and nuclear hormone receptors may have important clinical implications when interpreted alongside mechanistic data. The findings offer support to previous mechanistic studies which implicate P38 in the regulation of ER and PR. Turnover of ER is required for its activity, enabling it to respond to temporal fluctuations in oestrogen (oestradiol, E2) and/or the presence of tamoxifen. It has been shown that P38 phosphorylates ER at a specific site that demarcates the nuclear receptor for turnover via the ubiquitin system [26]. In contrast, P38 may have a role in maintaining the stability of PR [27]. Together with data showing that PR reprogrammes genomic ER binding to sites associated with favourable clinical outcome [28], this implies that P38 may confer a good clinical outcome and response to endocrine therapy by regulating nuclear receptors.

The current study also demonstrates that for patients with primary breast cancer treated with endocrine therapy for up to 5 years, high expression of p-P38 remains prognostic of good long-term survival. However, there was no significant difference in survival for the first 5–10 years for patients treated with endocrine therapy stratified by p-P38 status (high/low). During this period, endocrine therapy is likely to be the main determinant of clinical outcome in ER and/or PR positive disease.

In recent years, evidence from pivotal trials such as MA17R, ATTOM and ATLAS has shown that extended adjuvant endocrine therapy maintains its benefit for a longer period [29–31]. However, this is at the cost of loss of quality of life through side effects such as hot flushes, cumulative toxicity such as bone demineralisation, and extended risk of life threatening side effects such as thromboembolism.

In practice, whether to extend endocrine treatment is considered on a case-by-case basis. Guidance to predict the risk of relapse would be welcome in clinical practice and may include assessment of p-P38 using IHC on the primary tumour as a marker of long-term outcome. This hypothesis would need to be prospectively validated in a randomised clinical trial.

Despite the co-expression of p-P38 with ER and PR, the current dataset also demonstrates that the prognostic significance of P38 is maintained independently of biomarkers in current clinical use. This supports the concept of P38 as a tumour suppressor in breast cancer.

### Future directions

The current study validates existing mechanistic data and permits generation of new hypotheses, which can be explored with greater confidence in pre-clinical and clinical trials of primary breast cancer treatment. For example, clinical trials of P38 inhibitors in combination with adjuvant chemotherapy are supported by these data.

The predictive value of p-P38 expression by IHC on the relative risk of relapse following 5 years of adjuvant endocrine therapy will require further investigation with prospective clinical studies. This demonstrates the value of high quality prognostic data on the generation of new hypotheses with potential clinical utility.

## Conclusions

This study provides definitive evidence for the prognostic value of P38 in primary breast cancer and its molecular subtypes. The findings support a role for P38 as a tumour suppressor in breast cancer via upregulation of DNA repair, and provide hypothesis-generating information on the potential role of P38 in adjuvant therapy decision making.

## Additional files

Additional file 1: **Figure S1.** Western blotting results of pan-P38 and p-P38 expression in MCF-7 cell line lysate. **Figure S2.** Pan-P38 and p-P38 staining in breast cancer samples. (DOCX 463 kb)

## Abbreviations

ATF2: Cyclic-AMP dependent transcription factor 2
CI: Confidence interval
ER: Oestrogen receptor
HER2: Human epidermal growth factor receptor 2
HR: Hazard ratio
IHC: Immunohistochemistry
METABRIC: Molecular Taxonomy of Breast Cancer International Consortium
NPI: Nottingham prognostic index
OS: Overall survival
P38 MAPK: P38 mitogen activated protein kinase
p-P38: phosphorylated P38
PR: Progesterone receptor
TMA: Tissue microarray
VI: Vascular invasion
