# Supplementary material for: Co-expression of nuclear P38 and hormone receptors is prognostic of good long-term clinical outcome in primary breast cancer and is linked to upregulation of DNA repair
Source: BMC Cancer. 2018 Oct 23;18:1027. doi: 10.1186/s12885-018-4924-2 (PMC6199714; doi:10.1186/s12885-018-4924-2)
Supplement: Supplementary file 1 — Figure S1. Western blotting results of pan-P38 and p-P38 expression in MCF-7 cell line lysate. Figure S2. Pan-P38 and p-P38 staining in breast cancer samples. (DOCX 463 kb) [file 12885_2018_4924_MOESM1_ESM.docx]

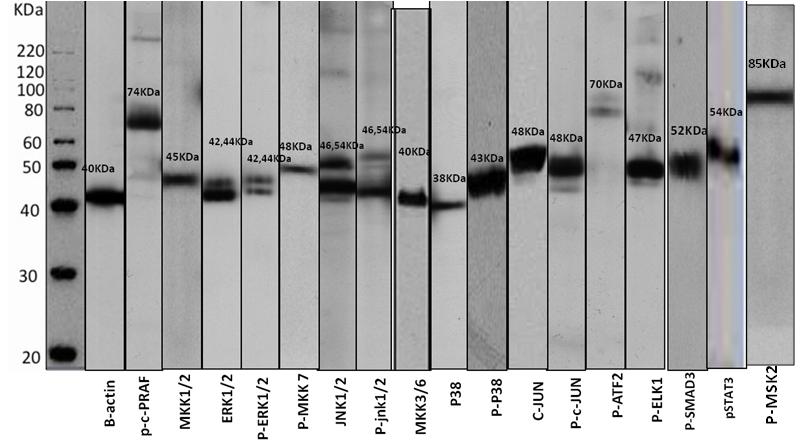

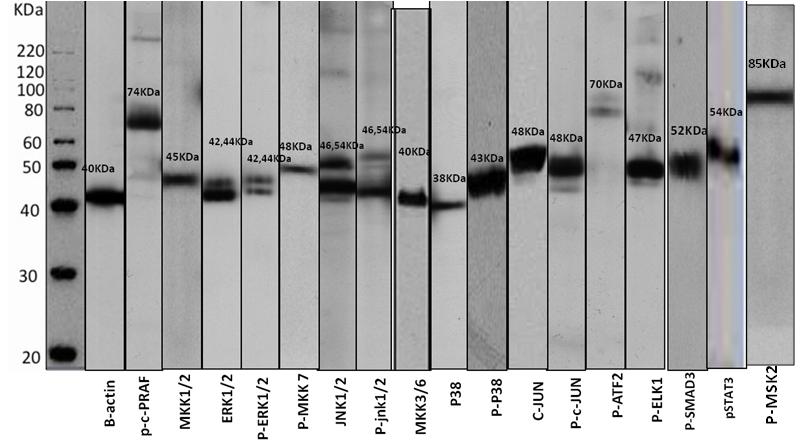


**Figure S1** Western blotting results of pan-P38 and p-P38 expression in MCF-7 cell line lysate.


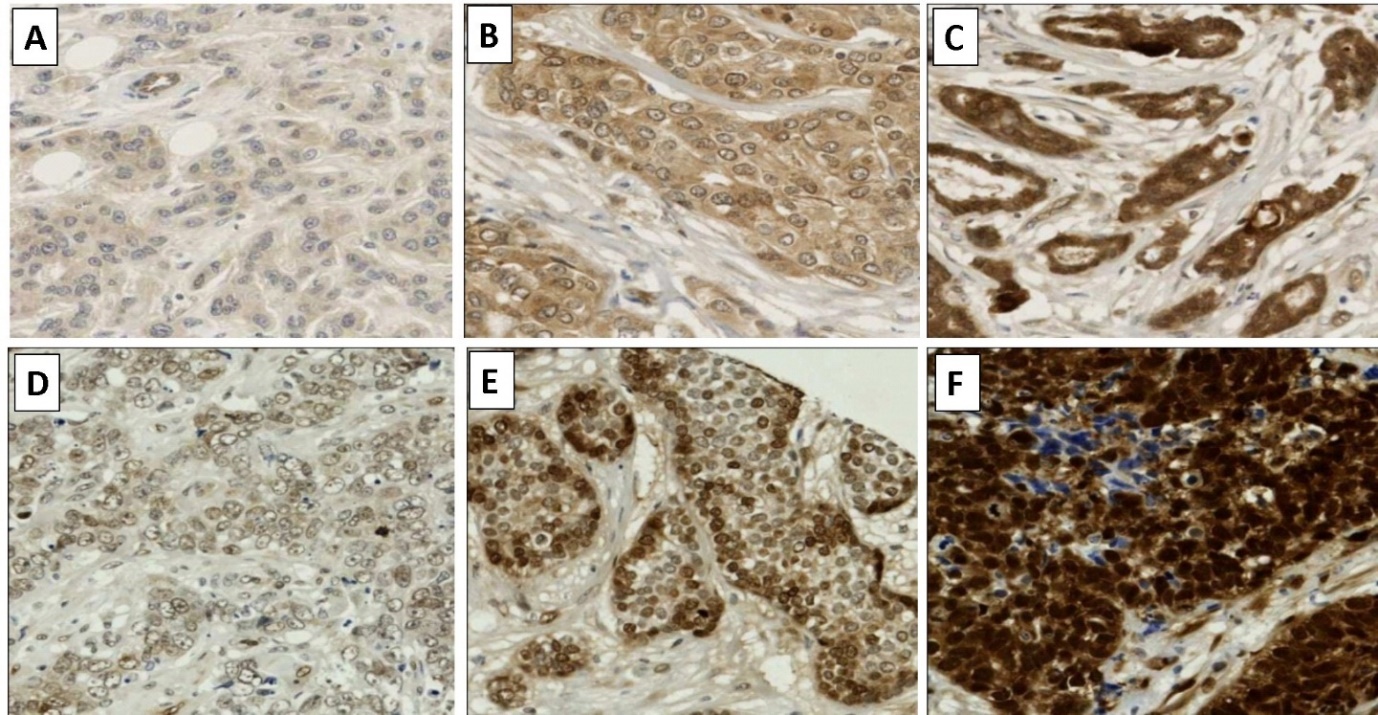


**Figure S2** Pan-P38 and p-P38 staining in breast cancer; Different intensities of staining were observed across samples. Representative images of weak, moderate and strong pan-P38 (A-C) and p-P38 (D-F), respectively, are shown. Photomicrograph magnification x200.
